# Impact of tracheostomy tube modalities on ventilatory mechanics: a bench study

**DOI:** 10.1186/s40635-024-00648-1

**Published:** 2024-07-08

**Authors:** Yann Combret, Margaux Machefert, Guillaume Prieur, Emeline Fresnel, Elise Artaud-Macari, Bouchra Lamia, Marius Lebret, Clément Medrinal

**Affiliations:** 1 Université Paris-Saclay, UVSQ, Erphan, 78000 Versailles, France; 2 Intensive Care Unit Department, Le Havre Hospital, Avenue Pierre Mendes France, 76290 Montivilliers, France; 3 Pulmonology Department, Le Havre Hospital, Avenue Pierre Mendes France, 76290 Montivilliers, France; 4 Physiotherapy Department, Le Havre Hospital, Avenue Pierre Mendes France, 76290 Montivilliers, France; 5 Kernel Biomedical, 18 Rue Marie Curie Bâtiment ANIDER, 76000 Rouen, France; 6 UR3830 GRHVN, Institute for Research and Innovation in Biomedicine (IRIB), Normandie Univ, UNIROUEN, 76000 Rouen, France; 7 Department of Pulmonary, Thoracic Oncology and Respiratory Intensive Care, CHU Rouen, 76000 Rouen, France; 8 Pulmonology, Respiratory Department, Rouen University Hospital, Rouen, France

**Keywords:** Cuff, High flow, Speaking valve, Tracheostomy, Weaning, Work of breathing

## Abstract

**Purpose:**

Tracheostomized patients often present with muscle weakness, altered consciousness, or swallowing difficulties. Hence, the literature is scarce regarding the challenging management of tracheostomy weaning. There is a need to strengthen the understanding of respiratory mechanisms with the different tracheostomy tube modalities that compose this weaning pathway. We aimed to evaluate the impact of these modalities on the work of breathing (WOB), total positive end-expiratory pressure (PEEPtot), and tidal volume (*V*_T_).

**Methods:**

With a three-dimensional (3D) printed head mimicking human upper airways, we added a tracheal extension, and pierced to allow insertion of a size 7.0 tracheostomy cannula. The whole was connected to an artificial lung. Three lung mechanics were simulated (normal, obstructive and restrictive). We compared five different tracheostomy tube modalities to a control scenario in which the tube was capped and the cuff was deflated.

**Results:**

A marginal difference was observed on the WOB within conditions with a slight increase + 0.004 [95% CI (0.003–0.004); *p* < 0.001] when the cuff was inflated in the normal and restrictive models and a slight decrease in the obstructive model. The highest PEEPtot that was reached was + 1 cmH_2_O [95% CI (1–1.1); *p* < 0.001] with high-flow therapy (HFT) with the cuff inflated in the obstructive model. We observed a statistically significant reduction in *V*_T_ [up to − 57 mL 95% CI (− 60 to − 54); *p* < 0.001] when the cuff was inflated, in both the normal and obstructive models.

**Conclusions:**

Our results support the use of conditions that involve cuff deflation. Intermediate modalities with the cuff deflated produced similar results than cannula capping.

## Background

Ventilatory weaning via tracheostomy is a common practice in intensive care units (ICU) for patients undergoing invasive mechanical ventilation [1–3]. This decision typically arises from challenging or prolonged ventilatory weaning scenarios. In France, tracheostomy weaning is recommended after two extubation failures or in cases of prolonged weaning [4]. Patients often present with muscle weakness, altered consciousness, or swallowing difficulties under such circumstances [5]. Despite well-described extubation criteria, literature on the management of ventilatory weaning in tracheostomized patients and decannulation criteria remains scarce and of low quality evidence [6–8].

On the pathway for tracheostomy weaning, tracheostomy tube modalities depend on the clinician’s judgment, which can also be influenced by institutional practices, the absence of tools for objective patient evaluation, and the limited understanding of the physiological impact of these modalities. Recent data advocate for cuff deflation and promotion of phonation to enhance patient communication, swallowing abilities, and shorten the time to decannulation [7, 9, 10]. Nevertheless, many tracheostomized patients will remain with the cuff inflated and an artificial nose for up to 18 days or more [9, 11]. Primary concerns raised by physicians are that cuff deflation would induce a loss of positive end-expiratory pressure (PEEP) that will reduce lung volume because of alveolar collapse, or that the use of a speaking valve could increase upper airway resistance and work of breathing, consequently increasing the risk of aspiration [10, 12]. To optimize ICU practices to promote cuff deflation and facilitate patient phonation, there is a need to enhance our understanding of respiratory mechanisms based on tracheostomy tube modalities. Hence, we aimed to evaluate the impact of different tracheostomy tube application methods on ventilatory mechanics and properties using an experimental bench mode. We hypothesized that the work of breathing (WOB), total positive end-expiratory pressure (PEEPtot), and tidal volume (*V*_T_) may vary depending on the modality of tracheostomy tube usage. Furthermore, we evaluated the airway's relative humidity (RH) and the fraction of inspired oxygen (FiO2).

## Methods

**Fig. 1:**
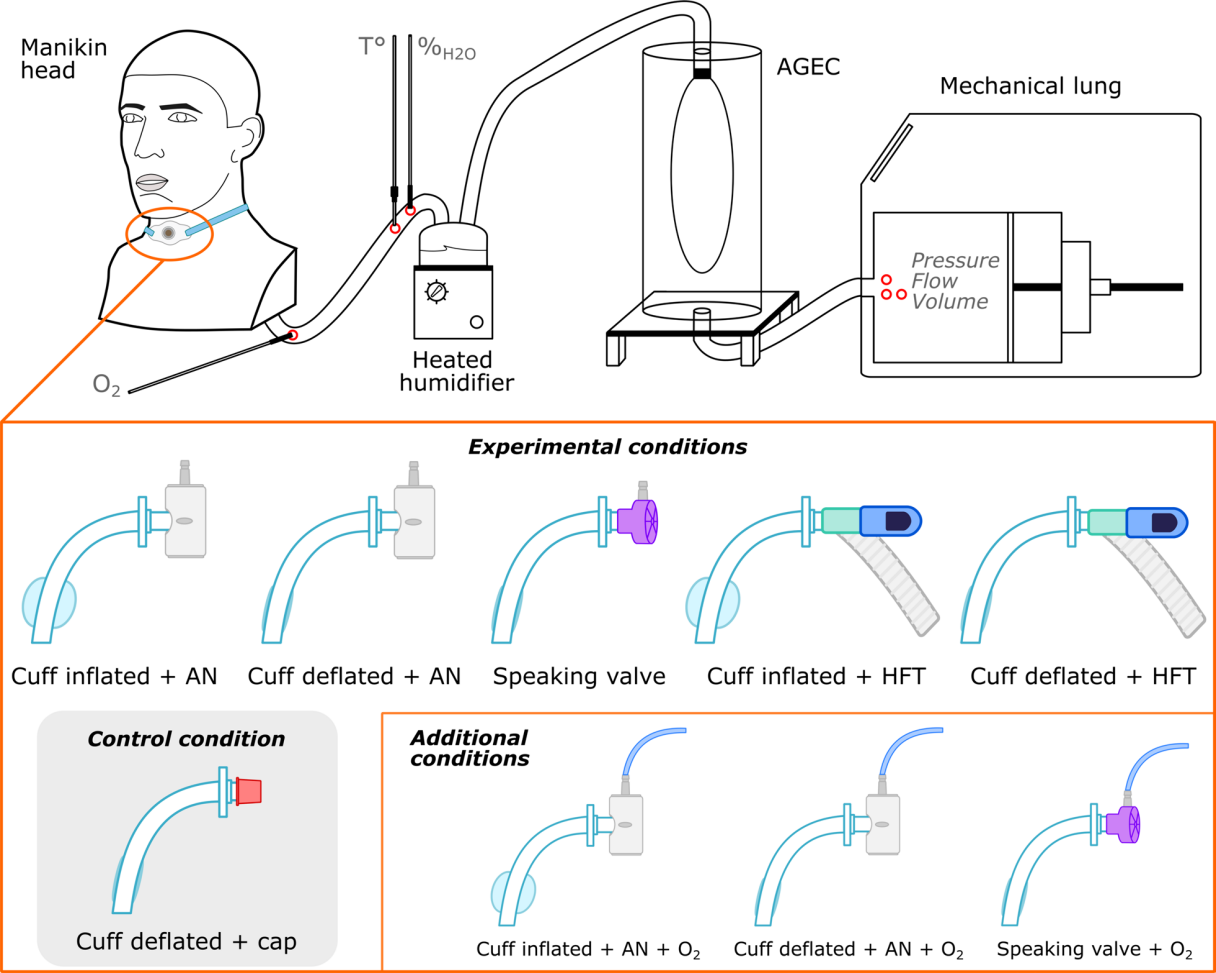
Experimental setup schematic (from left to right: realistic upper airway model head with tracheostomy module, heated humidifier, protective balloon (AGEC), ASL 5000 mechanical lung) and description of the experimental conditions. Red circles indicate the measurement sites of the main variables. AN: artificial nose, HFT: high flow therapy

We used a three-dimensional (3D) printed head mimicking human upper airways down to the trachea (KerNel Biomedical, France). The head model included a mouth with a seal, pliable components for the pharynx and trachea, and was enveloped in silicone skin, all mounted on a stable base. The airways had a dead space of 152 mL and a resistance of 2.4 cmH_2_O.s/L. The model's validity was established in a prior study [13]. A tracheal extension was added to the manikin head, pierced to allow insertion of a size 7.0 (inner diameter 7.0 mm, outer diameter 9.6 mm, length 80 mm) tracheostomy cannula (Portex Bivona Tracheostomy Tube, Smiths Medical, UK). The trachea was connected to an artificial lung (ASL 5000, IngMar Medical, USA) via a 22 mm diameter breathing circuit. To reproduce the hygrometric conditions typical of the lower airways, a heated humidifier (MR810, Fisher & Paykel Healthcare, New Zealand), was inserted in the circuit. The setup was finalized by adding a protective balloon (AGEC, IngMar Medical) to prevent contact between humidity and the internal parts of the mechanical lung. The total length of the breathing circuit sections between the manikin head and the artificial lung was 150 cm; compensation parameters were input into the ASL 5000 software (version 3.6) accordingly. Our experimental setup is presented in the Fig. 1.

Three lung mechanics were simulated on the artificial lung, by setting the airway resistance and thoraco-pulmonary compliance parameters according to data from the literature [14] as presented below. For each lung mechanics scenario, we calibrated the breathing dynamics to maintain a respiratory rate of 22 breaths per minute and a baseline tidal volume of 400 mL by modulating the strength of inspiratory effort for each lung condition. We simulated the three scenarios successively, producing 100 respiratory cycles in total for each lung mechanic.

Resistance and compliance were as follows:

Normal Model: 5 cmH_2_O.s/L and 60 mL/cmH_2_O.

Obstructive Model: 25 cmH_2_O.s/L and 60 mL/cmH_2_O.

Parieto-restrictive: 5 cmH_2_O.s/L and 30 mL/cmH_2_O.

### Experimental procedure

We evaluated five distinct tracheostomy setups against a baseline scenario where the tube was capped, and the cuff deflated. This baseline was deemed the control condition, as it is presumed to represent the most challenging phase for patients prior to decannulation.

These different configurations included the use of an artificial nose with both an inflated cuff and a deflated cuff, the application of a speaking valve with a deflated cuff, and the addition of high-flow therapy (HFT) with both inflated and deflated cuff conditions. HFT was administered through an Airvo2 (Fisher & Paykel Healthcare) device at 50 L/min, 37 °C and 21% oxygen fraction. Supplementary oxygen was delivered at a rate of 2 L/min with both artificial nose and speaking valve configurations, leading to three additional conditions tested. Each configuration was evaluated using the three simulated lung models.

### Data acquisition and statistical analysis

Pressure and flow data were acquired directly from the ASL 5000 software, at a sampling rate of 512 Hz. A calculation code (KerNel Biomedical, France) was then used to compute the following indicators on a cycle-by-cycle basis: WOB, in J/L, over the inspiratory phase, PEEPtot (in cmH_2_O), effective *V*_T_ (in mL). A hygrometry sensor (HMP110, Vaisala, Finland) and an oxygen sensor (PSR-11-917, Analytical Industries Inc., USA) were added at the distal end of the trachea. Temperature (in °C), relative (in %) and absolute (in mgH_2_O/L) humidity in the trachea and inspired oxygen fraction (in %) were measured and averaged over the duration of each simulation.

Quantitative data were expressed as mean (standard deviation) or median (interquartile range) depending on whether the distribution was normal or not. Normality of the distribution was assessed using Shapiro–Wilk test. We use the one-way ANOVA with Bonferroni correction. Comparisons were established to the Capping Condition, and expressed as mean differences and standard deviations or 95% confidence interval (CI). Tests were two-tailed and a value of *p* < 0.05 was considered statistically significant. Statistical analysis was performed using GraphPad X.

## Results

### Work of breathing

**Fig. 2:**
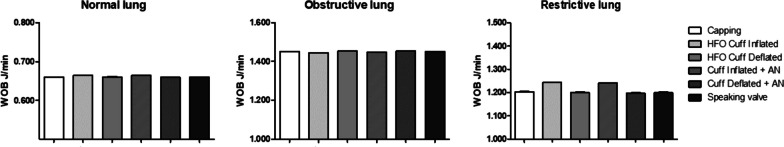
Absolute values of work of breathing (WOB) (in J/L) according to the lung model and the tracheostomy tube modality. Values are represented as mean and standard deviation

In the normal lung model, the mean WOB was 0.660 ± 0.002 J/L in the control condition (i.e., cannula capping). The conditions with the cuff deflated did not result in a notable change in the WOB (see Table 1). Inflation of the tracheostomy cuff statistically increased the WOB compared to the control condition but did not reach clinical relevance (see Table 1). For example, with the cuff inflated and HFT, the WOB was 0.664 ± 0.001 J/L, and with the cuff inflated alongside the use of an artificial nose, the WOB was 0.664 ± 0.002 J/L. In the restrictive lung model, deflating the cuff seemed to lead to a statistical decrease in WOB compared to the control condition, while inflating the cuff increased the WOB. The absolute values of WOB for the three models are presented in the Fig. 2.

**Table 1:** Mean differences and 95% confidence interval compared to the “canula capping” control condition for each experimental condition tested

Lung models	Compared to the ‘Cannula Capping’ condition	HFT with cuff inflated	HFT with cuff deflated	Artificial nose with cuff inflated	Artificial nose with cuff deflated	One way speaking valve
Normal	Work of breathing (J/L)	0.004 (0.003 to 0.004)	0 (− 0.001 to 0.001)	0.004 (0.003 to 0.004)	− 0.001 (0.001 to 0)	0 (− 0.001 to 0.001)
	PEEPtot (cmH_2_O)	1 (0.93 to 1.06)	0.10 (0.03 to 0.16)	0.23 (0.17 to 0.28)	− 0.08 (− 0.10 to − 0.02)	− 0.08 (− 0.10 to − 0.02)
	Tidal volume (mL)	− 57 (− 60 to − 54)	1 (− 1 to 4)	− 46 (− 49 to − 43)	8 (5 to 11)	7 (4 to 10)
Obstructive	Work of breathing (J/L)	− 0.009 (− 0.009 to − 0.008)	0.001 (0.001 to 0.001)	− 0.006 (− 0.006 to − 0.005)	0.001 (0.001 to 0.001)	0 (0 to 0)
	PEEPtot (cmH_2_O)	1 (1 to 1.10)	0.14 (0.11 to 0.17)	0.30 (0.27 to 0.33)	− 0.01 (− 0.03 to 0.01)	− 0.04 (− 0.06 to − 0.01)
	Tidal volume (mL)	− 43 (− 44 to − 43)	− 0.4 (− 1 to − 0.3)	− 35 (− 36 to − 35)	3 (2 to 4)	0.6 (− 0.0 to 1)
Restrictive	Work of breathing (J/L)	0.040 (0.030 to 0.040)	− 0.004 (− 0.005 to − 0.003)	0.037 (0.036 to 0.038)	− 0.007 (− 0.008 to − 0.006)	− 0.005 (− 0.006 to − 0.004)
	PEEPtot (cmH_2_O)	0.55 (0.37 to 0.73)	0.06 (0.12 to 0.24)	− 0.20 (− 0.40 to − 0.04)	− 0.20 (− 0.40 to − 0.04)	− 0.40 (− 0.50 to − 0.20)
	Tidal volume (mL)	− 8 (− 12 to − 3)	0.3 (4 to 5)	− 2 (− 6 to − 2)	6 (1 to 10)	10 (6 to 14)

HFT: high flow therapy; mL: milliliter; cmH_2_O: centime of water; PEEPtot: positive end expiratory pressure; J: joules

Conversely, in the obstructive lung model, WOB decreased in the conditions where the cuff was inflated, with HFT and with an artificial nose, compared to the control condition. HFT and artificial nose with the cuff inflated led, respectively, to a WOB of 1.400 ± 0.001 J/L and 1.500 ± 0.001 J/L.

### PEEPtot

**Fig. 3:**
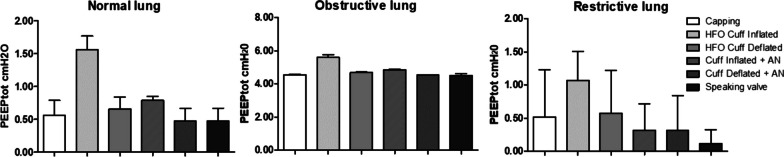
Absolute values of the total positive end-expiratory pressure (PEEPtot) (in cmH_2_O) a according to the lung model and the tracheostomy tube modality. Values are represented as mean and standard deviation

Figure 3 shows PEEPtot values under different conditions. HFT with cuff inflated significantly increased PEEPtot the most compared to capping for both the normal and obstructive models [respectively, + 1 cmH_2_O 95% CI (0.93–1.06); *p* < 0.001 and + 1 cmH_2_O 95% CI (1–1.10); *p* < 0.001]. For the restrictive model, cuff inflated with HFT increases PEEPtot by + 0.55 cmH_2_O 95% CI (0.37–0.76) (*p* < 0.001) compared to capping (see Table 1).

### Tidal volume

**Fig. 4:**
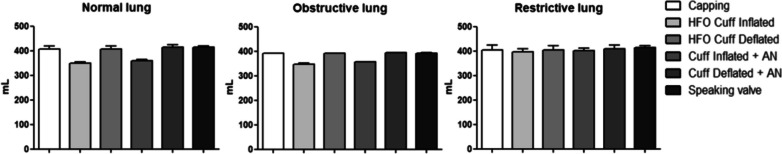
Absolute values of the effective tidal volume (*V*_T_) (in mL) according to the lung model and the tracheostomy tube modality. Values are represented as mean and standard deviation

We observed a significant reduction in *V*_T_ when the cuff was inflated, i.e., with HFT and with an artificial nose, across all three models (see Table 1). The decrease in *V*_T_ could reach − 57 mL 95% CI (− 60 to − 54) (*p* < 0.001) with HFT with cuff inflated compared to the cannula capping condition. The evolution of *V*_T_ according to the tracheostomy tube modalities is represented in Fig. 4.

### Relative humidity and oxygen supplementation

**Fig. 5:**
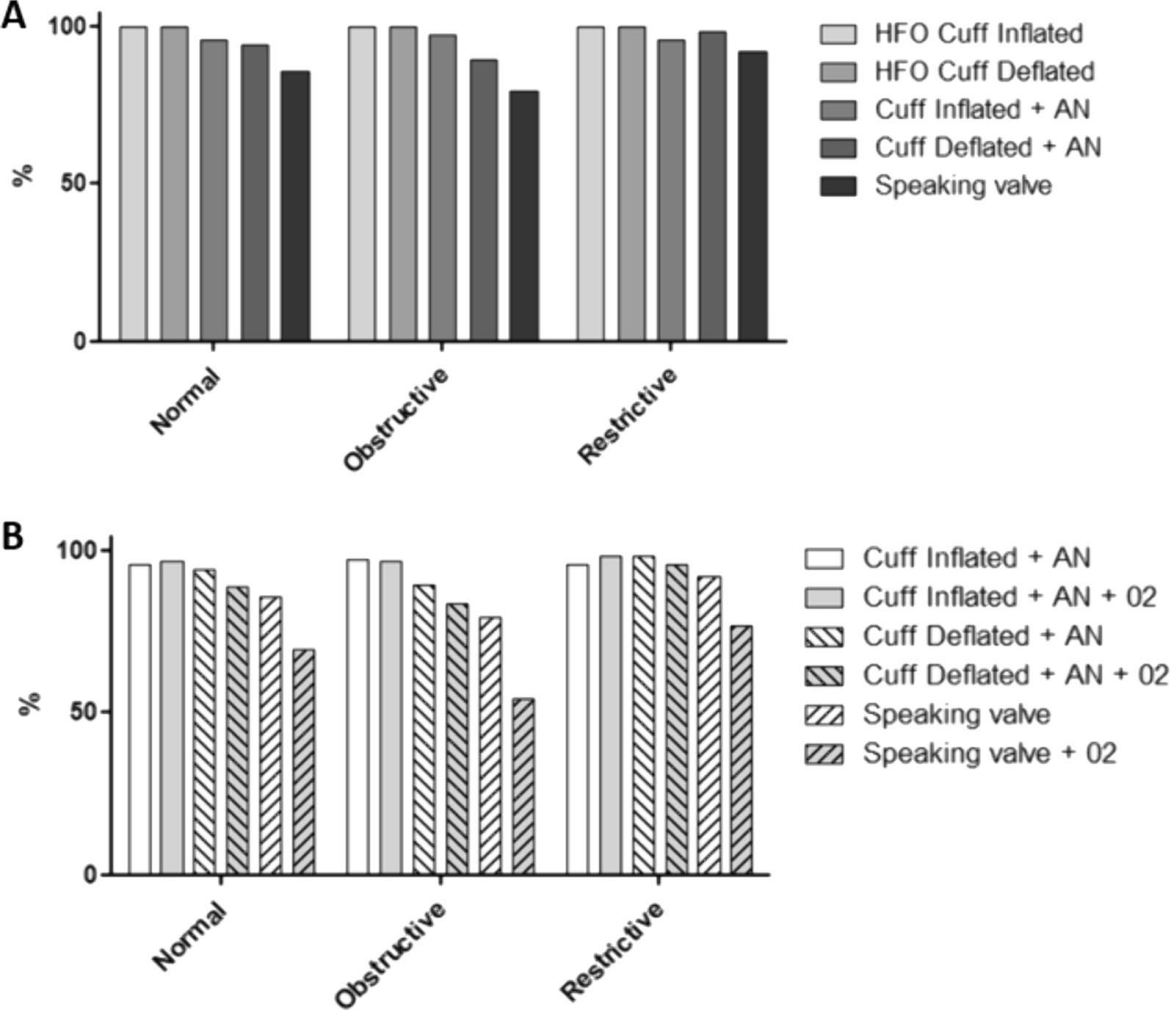
**A** Relative humidity resulting from tracheostomy tube modalities; **B** relative humidity resulting from either incorporating or not incorporating an oxygen flow of 2 L/min. Values are represented as mean

We observed in the Fig. 5 panel A that relative humidity was maintained with the use of an artificial nose, with a percentage ranging from 90% to 99%, depending on the lung models. Relative humidity was impacted by the use of the one-way speaking valve with a percentage ranging from 79.5% to 92%. The cannula capping condition was excluded from this analysis, because the air could not be humidified through natural airways in this bench study.

Panel B of Fig. 5 illustrates the variations in relative humidity resulting from either incorporating or not incorporating an oxygen flow of 2 L/min. The use of the speaking valve significantly affected relative humidity, with respective decreases of 16% in normal lung conditions, 25% in obstructive lung conditions, and 15% in restrictive lung conditions. The addition of 2 L/min of airflow further reduced the relative humidity.

The inspired oxygen fraction (FiO_2_) generated by administering an external flow of 2L/min was statistically increased with the use of a one-way speaking valve compared to the cuff inflated with an artificial nose condition [respectively, 46% 95% CI (42.5–49.5) vs. 26.7% 95% CI (22.9–30.5); *p* < 0.001]. Inflated or deflated cuff did not statistically influence FiO_2_ with an artificial nose.

## Discussion

This bench study provided the following observations: (i) in the normal and restrictive models, conditions with an inflated cuff led to a slight increase in ventilatory work compared to the capping condition which is the last step on the decannulation pathway [15]. However, in the obstructive model, ventilatory work was minimally increased by the cuff deflated conditions. (ii) Adding HFT to a cuff-inflated tracheostomy tube increased PEEPtot by up to 1 cmH_2_O compared to the capping condition. (iii) Across all pulmonary models, the inflated cuff resulted in a reduction in *V*_T_ by up to − 57 mL per breath.

In our experimental conditions, the observed results strengthen the rationale for cuff deflation. The differences observed on the WOB compared to cannula capping, although sometimes statistically significant, appear clinically minimal and may not discourage cuff deflation considering the other benefits (e.g., improved swallowing or phonation) expected. The only exception was noted in the obstructive lung model, with an increase in WOB occurring with cuff deflation. One explanation could be that the decrease in expiratory resistance while the cuff is inflated in such circumstance might be beneficial for patients with obstructive diseases [16, 17]. However, it is important to note that our model does not replicate vocal cords and their physiological impact on patient respiratory mechanics. In addition, numerous clinical arguments support cuff deflation and the use of the speaking valve to facilitate expectoration, swallowing, and lung recruitment [1, 2, 7, 9–12].

Interestingly, the use of HFT did not produce the expected effects on WOB or the generation of PEEPtot. The clinical hypotheses were that HFT could serve as a substitute for mechanical ventilation by reducing WOB, maintaining PEEP effects, and increasing *V*_T_ [18, 19]. However, our results do not corroborate these hypotheses, with minimal PEEP effects (+ 1 cmH_2_O at best) and decreased *V*_T_. Nevertheless, our results indicate that HFT provides the best humidification and the highest temperature, while the speaking valve significantly reduces relative humidity. These findings are further accentuated when oxygen is added. Indeed, the mechanism of oxygen administration on the speaking valve traps oxygen behind the one-way membrane and exacerbates the drying of inspired gases. These results are confirmed by the differences in FiO_2_ found for the same flow rate, with FiO_2_ of 46% 95% CI (42.5–49.5) for 2 L/min of O_2_.

Our study had several limitations, including the lack of air warming and humidification typically provided by the upper airway mucosa, notably the nasal turbinates. This could have potentially affected our findings related to relative humidity in conditions where the cuff was deflated. In addition, our model did not incorporate vocal cords, which precluded us from considering the permeability of a patient's upper airways in our analysis. We also did not compare the different existing tracheostomy tube models or the possibility of incorporating fenestrated tubes to facilitate phonation. Given that our results indicated minimal clinical differences between the most challenging scenario (cannula capping) and the other conditions tested, we would expect that the subtle variations inherent to different tracheostomy tube models would likely have little impact on patient outcomes. Last, the minimal variability in the WOB across different tracheostomy modalities could be attributed to the inspiratory effort being specifically calibrated for each lung model. If the respiratory effort settings on our models were more adaptive, a greater variability in WOB might have been anticipated depending on the conditions. Although the controlled conditions of our experimental model enhance the reliability of our findings, the lack of variability could limit how directly they can be applied to real-world clinical settings. Notably, some statistical differences we observed, particularly in the WOB, are likely to have limited clinical significance. Hence, these results should be cautiously interpreted and may need adaptation for actual clinical environments, considering factors beyond our experimental scope.

Our results support the use of conditions that involve cuff deflation. We also noted that intermediate modalities, such as using an artificial nose or a speaking valve with the cuff deflated, yielded results that were very close to, if not indistinguishable from, those observed with cannula capping. Considering the risk of airway dryness, the reduction in the number of days before decannulation, and in the absence of contraindications (glottic control disorders and upper airway obstruction), the choice between capping and speaking valve may look trivial and should be more discussed among the healthcare staff.

In conclusion, our innovative model allows us to provide arguments for cuff deflation. The differences between intermediate conditions and capping are minimal and encourage clinicians to reduce the number of steps before attempting capping. The results of this bench study need to be validated with clinical data.

## Data Availability

Clément Medrinal had full access to all the data in the study and takes responsibility for the integrity of the data and the accuracy of the data analysis. The study specific summary data can be obtained from the corresponding author: Clément Medrinal; medrinal.clement.mk@gmail.com.

## Abbreviations

CI: Confidence interval
FiO_2_: The inspired oxygen fraction
HFT: High flow therapy
ICU: Intensive care unit
PEEPtot: Positive end-expiratory pressure
*V*_T_: Tidal volume
WOB: Work of breathing
